# DNA Polymerase Iota Promotes Esophageal Squamous Cell Carcinoma Proliferation Through Erk-OGT-Induced G6PD Overactivation

**DOI:** 10.3389/fonc.2021.706337

**Published:** 2021-07-20

**Authors:** Zhenzi Su, Aidi Gao, Xiaoqing Li, Shitao Zou, Chao He, Jinchang Wu, Wei-Qun Ding, Jundong Zhou

**Affiliations:** ^1^ Department of Radiation Oncology, The Affiliated Suzhou Hospital of Nanjing Medical University, Suzhou, China; ^2^ Suzhou Cancer Center Core Laboratory, The Affiliated Suzhou Hospital of Nanjing Medical University, Suzhou, China; ^3^ The Second Affiliated Hospital of Xuzhou Medical University, Xuzhou, China; ^4^ Department of Pathology, University of Oklahoma Health Science Center, Oklahoma City, OK, United States

**Keywords:** DNA polymerase iota, Erk signaling pathway, ESCC, G6PD activity, tumor proliferation

## Abstract

Esophageal squamous cell carcinoma (ESCC) is one of the most lethal cancers with rapid progression and a high mortality rate. Our previous study demonstrated that DNA polymerase iota (Pol ι) is overexpressed in ESCC tumors and correlates with poor prognosis. However, its role in ESCC proliferation remains obscure. We report here that Pol ι promotes ESCC proliferation and progression through Erk- O-GlcNAc transferase (OGT) regulated Glucose-6-phosphate dehydrogenase (G6PD) overactivation. Cell clonogenic ability was assessed by colony formation assay. Cell proliferation was assessed by EdU incorporation assay. Our transcriptome data was reanalyzed by GSEA and validated by analysis of cellular metabolism, G6PD activity, and cellular NADPH concentration. The level of Pol ι, OGT, G6PD and O-GlcNAcylation in ESCC cells and patient samples were analyzed. The MEK inhibitor PD98059 was applied to confirm OGT expression regulation by the Erk signaling. The G6PD inhibitor polydatin was used to examine the role of G6PD activation in Pol ι promoted proliferation. We found that Pol ι promotes ESCC proliferation. It shunted the glucose flux towards the pentose phosphate pathway (PPP) by activating G6PD through OGT-promoted O-GlcNAcylation. The expression of OGT was positively correlated with Pol ι expression and O-GlcNAcylation. Notably, elevated O-GlcNAcylation was correlated with poor prognosis in ESCC patients. Pol ι was shown to stimulate Erk signaling to enhance OGT expression, and the G6PD inhibitor polydatin attenuated Pol ι induced tumor growth *in vitro* and *in vivo*. In conclusion, Pol ι activates G6PD through Erk-OGT-induced O-GlcNAcylation to promote the proliferation and progression of ESCC, supporting the notion that Pol ι is a potential biomarker and therapeutic target of ESCC.

## Introduction

Esophageal cancer is recognized as the sixth leading cause of cancer death worldwide with a poor 5-year survival rate of less than 20% (1, 2). Esophageal squamous cell carcinoma (ESCC) is the predominant form of esophageal cancer in the world (3–5). In China, it ranks the fourth of estimated cancer deaths (6). The survival rate of early-stage ESCC can be greatly improved by surgical treatment. Unfortunately, most ESCC patients were diagnosed at an advanced stage with rapid progression and poor prognosis (7). Thus, it is urgent to understand how ESCC progresses in order to improve its clinical outcomes.

DNA polymerase iota (Pol ι) belongs to the Y-family DNA polymerase and participates in translesion DNA synthesis (TLS) (8). Pol ι (product of the *POLI* gene) was identified as the second homolog of yeast *Rad30* gene in human (9). Although Pol ι is well-recognized for its function of bypassing DNA lesions during replication (8), recent studies suggested that Pol ι is involved in the progression of various types of cancer. Overexpression of Pol ι was found in breast cancer (10, 11), bladder cancer (12), glioma (13), and ESCC (14). Furthermore, elevated Pol ι activates the Erk and JNK signaling pathway, contributing to invasion, metastasis and poor prognosis of ESCC (15, 16). However, whether Pol ι plays a role in ESCC proliferation remains unclear.

One of the main overactivated metabolic pathways during rapid cancer progression is the pentose phosphate pathway (PPP), which regulates the production of the nucleotides structure component ribose-5-phosphate and nicotinamide adenine dinucleotide phosphate (NADPH) (17–19). Activation of the PPP renders tumor cells an advantage for proliferation and development (20–22). Glucose-6-phosphate dehydrogenase (G6PD) serves as a pacemaker of PPP due to its activity to shunt glucose flux to PPP and catalyze the first and rate-limiting step of PPP (17). Mounting evidence indicated that increased glucose flux towards PPP and overactivation of G6PD are common in many cancer types, including clear cell renal cell carcinoma, hepatocellular carcinoma, colorectal cancer, prostate cancer, and ESCC (23–27). It is known that G6PD overactivation in tumor cells is regulated at the transcriptional or posttranslational level (17, 21, 22, 27–29). Recent evidence suggested that OGT promotes O-GlcNAcylation of G6PD, and this process is critical for G6PD activation and tumor progression (30). However, it remains unclear how G6PD and PPP are overactivated in ESCC and whether Pol ι is involved in their regulation.

In this study, Pol ι was found to promote ESCC proliferation, resulting from activated G6PD that redirected glucose flux towards PPP. Mechanistically, we found that Pol ι activates G6PD through Erk-OGT-induced O-GlcNAcylation. This novel finding, along with our previous reports (14–16), indicates that Pol ι is a potential new biomarker and therapeutic target of ESCC.

## Materials and Methods

### Cell Lines and Cell Culture

Human ESCC cell lines, TE-1 and KYSE-150, were obtained from the Shanghai Cell Bank (Shanghai, China). The stable Pol ι downregulated KYSE-150 cells were previously generated (15). For stable Pol ι overexpression TE-1 cells generation, the human *POLI* gene was amplified and cloned into the lentivirus vector LV5 (GenePharma, Suzhou, China) for virus production. TE-1 cells were infected with lentivirus containing control plasmid or *POLI* gene and selected by Puromycin (Sigma-Aldrich, St. Louis, MO, USA). TE-1 cells were cultured in DMEM medium and KYSE-150 cells were cultured in RPMI-1640 medium. All the aforementioned media (Hyclone, Logan, UT, USA) were supplemented with 10% fetal bovine serum (FBS, Clark Bioscience, VA, Richmond, USA). Cells were incubated under standard conditions (5% CO_2_ and 37°C) in a humidified atmosphere.

### Colony Formation Assay

TE-1 and KYSE-150 cells were seeded at a density of 500 or 1,000 cells per well in 6-well plates, respectively. After 7-10 days of incubation under standard conditions, colonies were stained with the Wright-Giemsa staining kit (Nanjing JianCheng Technology, Nanjing, China). Colonies that contain more than 50 cells were counted. In the drug-treated group, cells were exposed to different concentrations of polydatin (Sigma-Aldrich, St.Louis, USA) after being attached overnight.

### EdU Cell Proliferation Assay

The EdU incorporation assay of different cell lines was performed using a BeyoClick™ EDU Cell Proliferation Kit with TMB (Beyotime Biotechnology, Shanghai, China) according to the manufacturer’s instructions. In brief, TE-1 and KYSE-150 cells were seeded in 96-well plates at a density of 1,000 cells per well. Cells were incubated with 10 μM EdU for 2 h at 37 °C, followed by fixation with 4% paraformaldehyde and permeabilization with 0.3% Triton X-100. Subsequently, 50 μL of Click reaction buffer containing biotin was added to each well. After incubation for 30 min at room temperature in the dark, Streptavidin-HRP working solution was added and incubated for another 30 min at room temperature. Then, cells were washed three times with PBS. After 5 min developing with the TMB developing buffer, the absorbance was measured at 630 nm using a microplate reader (Thermo Scientific, Rochester, NY, USA).

### Cellular Metabolism Rate Assay

Real-time ATP rate assay was performed using the Seahorse Bioscience XFp Analyzer (Agilent, Santa Clara, CA, USA). By measuring extracellular acidification rate (ECAR) and oxygen consumption rate (OCR), the fractions of ATP produced from mitochondrial oxidative phosphorylation and glycolysis can be distinguished. Briefly, 1 × 10^4^ cells were seeded in each well of Seahorse XFp Microplate. After cell attachment, the cultural media was changed by unbuffered assay medium containing 10 mM glucose, 1mM pyruvate, and 2 mM glutamine. Cells were then incubated in a non-CO_2_ incubator for 60 min at 37 °C. A baseline measurement was first performed, followed by sequential injection of 1.5 μM oligomycin and 0.5 μM each of Rotenone and Antimycin A mixture. Results were analyzed using the Real-Time ATP Rate Assay Report Generator.

### G6PD Activity Assay

The G6PD activity was measured using the G6PD Activity Assay Kit (Beyotime Biotechnology). Briefly, 1 × 10^5^ TE-1 and KYSE-150 cells or 10 mg tumor tissues were harvested and lysed with G6PD extracting solution at 4°C. Fifty μL protein sample and 50 μL of the G6PD test solution were successively added to each well of a 96-well plate. After incubating at 37°C for 10 min in the dark, the absorbance was measured at 450 nm using a microplate reader (Thermo Scientific). Protein concentration, determined by the BCA protein quantification kit (Beyotime Biotechnology), was used for normalization.

### NADPH Concentration Assay

The NADPH concentration in cells was measured according to the manufacture’s protocol using NADPH Assay Kit (Beyotime Biotechnology). Briefly, 1 × 10^5^ TE-1 and KYSE-150 cells were harvested and lysed with the extracting buffer at 4°C. Then, the protein sample was incubated at 60°C for 30 min to decompose NADP^+^. Fifty μL protein sample and 50 μL working solution were successively added to each well of a 96-well plate. After incubating at 37°C for 10 min in the dark, 10 μL developing buffer was added into each well and incubated for another 10 min. The absorbance was then measured at 450 nm using a microplate reader (Thermo Scientific). Protein concentration, determined by the BCA protein quantification kit (Beyotime Biotechnology), was used for normalization.

### RNA Isolation and Quantitative Real-Time PCR

Total RNA was isolated using TRIzol Reagent (Sigma-Aldrich). The RNA concentrations were determined using the NanoDrop2000 (Thermo Scientific). Total RNA (1 μg) was reverse-transcribed into cDNA in a 20 μL reaction mixture using the RevertAid First Strand cDNA Synthesis Kit (Thermo Scientific). Quantitative real-time PCR analyses were performed using the StepOne Plus instrument (Applied Biosystems, Rochester, NY, USA). The primers for human *POLI*, *OGT*, and *ACTB* were as follows: *POLI*, Forward: 5’-ACTTTCTGCGGTGACTGTGT-3’, Reverse: 5’-TACATGGCTTCCCGCATCTC-3’; *OGT*, Forward: 5’-GCTCACTTGCTTAGGTTGTCTT-3’, Reverse: 5’-GCCGCTCTAGTTCCATTGTG-3’; *ACTB*, Forward: 5’-CACCATTGGCAATGAGCGGTTCC-3’, Reverse: 5’-GTAGTTTCGTGGATGCCACAGG-3’. Relative *POLI* and *OGT* mRNA expression levels were calculated using the 2^-ΔCt^ method and normalized to *ACTB* expression levels.

### Western Blot Analysis

Cells were harvested and lysed with M-PER lysis buffer (Thermo Scientific) containing protease inhibitor cocktail and phosphatase inhibitor cocktail (Beyotime Biotechnology) for 30 min at 4°C. Protein concentration was determined using the BCA protein quantification kit (Beyotime Biotechnology). Equal amounts of the proteins were separated by SurePAGE™ precast gels with a linear gradient between 4%-20% (GenScript, Nanjing, China) and transferred to PVDF membranes (Millipore, Billerica, MA, USA) by eBlot^®^ L1 protein transfer system (GenScript). After blocking with 5% non-fat milk, the membranes were incubated with primary antibodies against β-actin (Beyotime Biotechnology), Pol ι (Proteintech, Rosemont, IL, USA), G6PD (Abcam, Cambridge, MA, USA), OGT (Proteintech), O-GlcNAc (Invitrogen Life Technologies, Carlsbad, CA, USA), Erk and p-Erk (Cell Signaling Technology, Danvers, Massachusetts, USA) at 4°C overnight. After washing with TBST three times, the membranes were incubated with HRP-conjugated anti-mouse or anti-rabbit secondary antibody (MultiSciences, Hangzhou, China). High-sig ECL Western Blotting Substrate (Tanon, Shanghai, China) was applied for band visualization. Images of the protein bands were collected by Tanon-5200 Chemiluminescent Imaging System (Tanon). β-actin expression was served as a loading control.

### Immunoprecipitation (IP) Assay

Cells were collected and lysed using RIPA lysis buffer (Beyotime Biotechnology) containing protease inhibitors for 30 min at 4°C. The protein was incubated with anti-G6PD and anti-IgG antibodies at 4°C overnight. Then Protein A/G agarose beads (Abcam) was added to each tube. After incubation again at 4°C for 3 h, the beads were collected by centrifugation at 2,000 x g for 2 min. Subsequently, the beads were washed with IP wash buffer three times, followed by adding 40 μL 2× loading buffer and denaturing at 100°C for 5 min. Western blot was then performed. The anti-O-GlcNAC antibody (RL2, Thermo Scientific) was used to test the O-GlcNAcylation of G6PD.

### Dual-Luciferase Reporter Assay

A predicted OGT promoter, -2,000 to +500 bp, was acquired from the NCBI RefSeq database and cloned into the pGL4 plasmid. The pGL4-OGT and pRL-TK were transfected into cells with a ratio of 10:1 for 48 h. Then the OGT promoter activity was examined by Dual-Luciferase Reporter Assay (Promega, Madison, WI, USA). In brief, cells were seeded in 6-well plates at a density of 1 × 10^5^ cells per well and incubated overnight. The pGL4-OGT plasmid was transfected into the corresponding cells using the Lipofectamine 3000 (Invitrogen, USA). Cells were collected and lysed in the 1× Passive Lysis Buffer (PLB, Promega, USA) at ambient temperature for 20 min. 20 μL lysate was transferred into the tube containing 100 μL Luciferase Assay Buffer II (LAR II, Promega). After mixing 3 times, the tube was placed in the GloMax 20/20 Luminous detector (Promega) and recorded the measurement. Then, 100 μL Stop & Glo Reagent (Promega) was added into the same tube and recorded the second measurement. Results of triplicate transfections were combined to evaluate OGT promoter luciferase activity.

### Immunohistochemistry (IHC)

Tumor tissues were paraffin-embedded and sectioned. For IHC analysis, the sections were dewaxed, hydrated, and heat-treated using sodium citrate, pH 6.0. Sections were blocked with 5% BSA at 37°C for 1 h, followed by incubation with primary antibody against Pol ι (Abcam), OGT, O-GlcNAcylation at 4°C overnight. After washing with PBS three times, the HRP-conjugated anti-mouse/rabbit secondary antibody was used to incubate the sections for 1 h at 37°C. Sections were developed with a DAB kit (Cwbiotech, Beijing, China). Hematoxylin was used for counterstaining. Then the sections were washed and mounted.

The expression of Pol ι, OGT, and O-GlcNAcylation was scored by two pathologists. The staining density was scored: 1 (<25%), 2 (26%-50%), 3 (51%-75%) and 4 (>75%). The staining intensity was scored: 1 (negative or weakly positive), 2 (positive), and 3 (strongly positive). The final score for each section was calculated by multiplying the scores of the density and intensity.

This study was approved by the Institutional Ethics Committee of Nanjing Medical University. Human samples were obtained from The Affiliated Suzhou Hospital of Nanjing Medical University (Jiangsu, China) with informed consent.

### MEK Inhibitor Assay

TE-1 cells were seeded at a density of 1 × 10^5^ cells per well in a 6-well plate. After incubation overnight at 37 °C, cells were treated with different concentrations of PD98059 for 24 h at 37 °C. Then cells extracts were blotted with Erk and p-Erk Antibody.

### Xenograft Studies

For the *in vivo* xenograft study, 6-8 weeks old female BALB/C nude mice were obtained from Shanghai SLAC Laboratory Animal Co. Ltd. (Shanghai, China). 1 × 10^7^ cells suspended in 100 μL normal saline were injected subcutaneously into nude mice at the left groin. The tumor volume was calculated by the 4/3 × π × [(long diameter/2) (short diameter/2)^2^] formula. When the average tumor size grew up to 300 mm^3^, the nude mice were divided into three groups (n = 6 for each group). In the treatment group, the mice were intraperitoneally injected with 5 mg/kg polydatin dissolved in normal saline every other day. Mice in untreated groups were administrated with the same volume of normal saline containing DMSO as control. The animal experiment was approved by the Ethics Committee of the Nanjing Medical University.

### Statistical Analysis

SPSS 19.0 software (IBM, Chicago, IL, USA) was used for Statistical analysis. All data were presented as mean ± standard deviation (SD). Differences between two groups were evaluated by the Student t-test. Differences among more groups were analyzed by one-way ANOVA. Spearman correlation was used to analyze the correlation between two genes expression. Statistical significance was considered to be a *P*-value < 0.05.

## Results

### Pol ι Promotes ESCC Colony Formation and Cell Proliferation *In Vitro*

**Figure 1:**
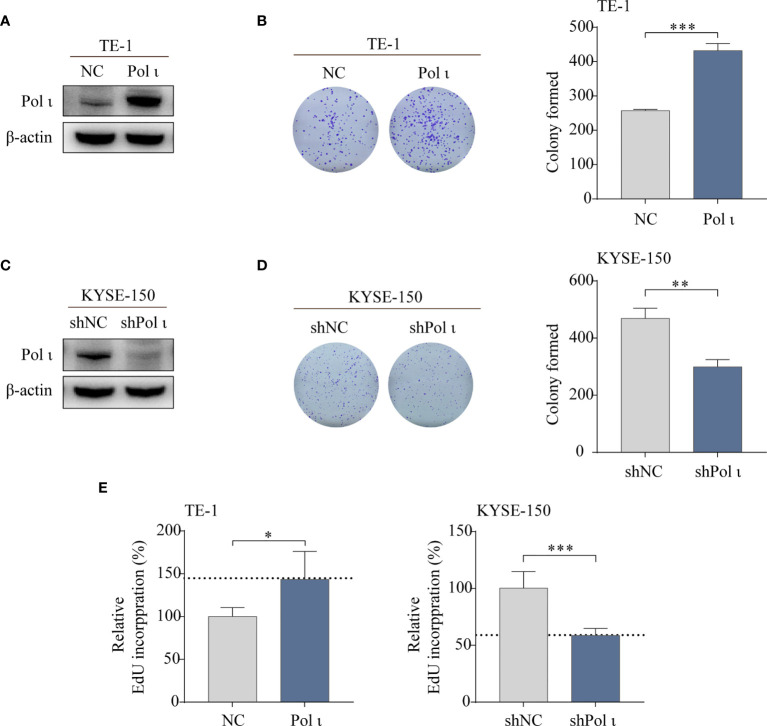
Pol ι promotes ESCC cell proliferation in *vitro.* **(A**, **C)** Differentially expressed Pol ι was confirmed by Western blot analysis in two ESCC cell lines with β-actin level as an internal control. The colony formation assay of TE-1 **(B)** and KYSE-150 cells **(D)**. **(E)** The EdU incorporation assay, results of NC or shNC cells were served as control and the results of Pol ι up-or down-regulated cells were presented relative to control. *P < 0.05, **P < 0.01, ***P < 0.001.

To investigate the influence of Pol ι on the proliferation of ESCC cells, Pol ι overexpressed TE-1 cells and downregulated KYSE-150 cells were used in this study. The expression of Pol ι in these cell lines was confirmed by Western blot (**Figures 1A, C**). We then performed colony formation assay and EdU incorporation assay for these cells. The results of the colony formation assay indicated that Pol ι overexpression strengthened the clonogenic abilities of TE-1 and KYSE-150 cells in comparison with the control group (**Figures 1B, D**). Similar results were observed in the EdU incorporation assay. As shown in **Figure 1E**, overexpression of Pol ι enhanced the proliferation of TE-1 cells compared to the control cells (*P* < 0.01), whereas only half of EdU incorporated in Pol ι downregulated KYSE-150 cells.

Taken together, these results indicated that Pol ι promotes ESCC cell colony formation and proliferation.

### Pol ι Induces Metabolic Transition by Enhancing G6PD Activity

**Figure 2:**
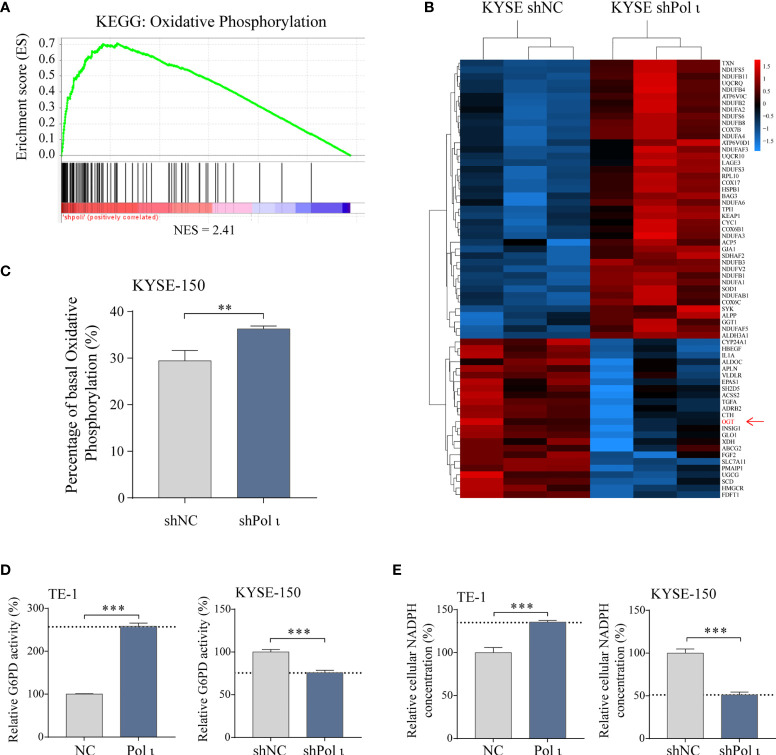
Pol ι induces metabolic transition. **(A)** RNA transcriptome data was reanalyzed using Gene Set Enrichment Analysis. Genes involved in the oxidative phosphorylation pathway were found enriched in Pol ι downregulated KYSE-150 cells. NES = 2.41, *P* < 0.001, FDR-q < 0.001. **(B)** heatmap of differentially expressed genes that participate in cellular metabolism. **(C)** rate of cellular metabolism tested by Seahorse analyzer using Real-Time ATP rate assay kit. Total ATP production, the sum of ATP generated from oxidative phosphorylation and Glycolysis, was considered 100%. The ATP production of each metabolic pathway was calculated by oxygen consumption rate (OCR) and extracellular acidification rate (ECAR) after serial injection of oligomycin (1.5 μM) and a mix of rotenone and antimycin A (0.5 μM each). Relative G6PD activity **(D)** and cellular NADPH concentration **(E)** in Pol ι differentially expressed TE-1 and KYSE-150 cells. **P < 0.01, ***P < 0.001.

To unveil the underlying mechanisms of Pol ι induced proliferation of ESCC cells, Gene Set Enrichment Analysis (GSEA, v4.1.0, MSigDB 7.2) (31, 32) was performed to reanalyze our transcriptome data (15) in Pol ι downregulated KYSE-150 shPol ι cells versus control KYSE-150 shNC cells. As shown in **Figure 2A**, many upregulated genes were enriched in the oxidative phosphorylation pathway, including subunits of NADH dehydrogenase (DUFA), ubiquinone oxidoreductase and Cytochrome c oxidase (**Figure 2B**). On the other hand, many downregulated genes were involved in other metabolism pathways, such as glycolysis or lipid metabolism. Of note, O-linked N-acetylglucosamine (GlcNAc) transferase (OGT), the key regulator of G6PD activity, was found to be downregulated in KYSE-150 shPol ι cells (**Figure 2B**).

We next applied cellular metabolism rate analysis to evaluate whether a metabolic transition was triggered in the wake of Pol ι-knocking down-induced upregulation of oxidative phosphorylation related genes. As expected, KYSE-150 shPol ι cells exhibited higher oxidative phosphorylation capacity, comparing to control cells (**Figure 2C**). Furthermore, cellular metabolism rate analysis also indicated that oxidative phosphorylation is similar but not reduced in Pol ι upregulated cells comparing with control cells (**Figure S1**). These results suggested that Pol ι may play a key role in the directional control of glucose flux.

**Figure 3:**
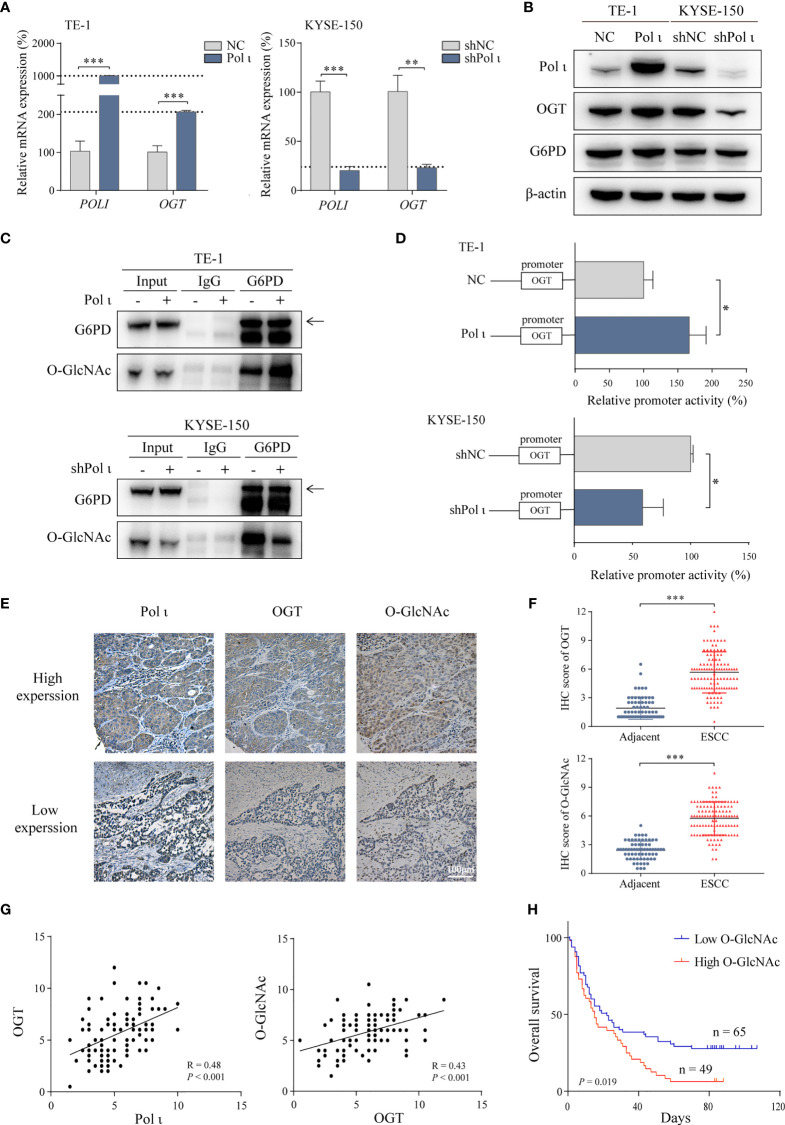
Pol ι activates G6PD through OGT-promoted O-GlcNAcylation. **(A)** The relative mRNA level of *POLI* and *OGT* in Pol ι differentially expressed TE-1 and KYSE-150 cells. **(B)** the protein level of Pol ι, OGT and G6PD. **(C)** O-GlcNAcylation of G6PD was detected after G6PD immunoprecipitation in ESCC cells. **(D)** the promoter of OGT (-2000 to +500 bp) was cloned into pGL-4 vector. The pGL4-OGT and internal control reporter vector pRL-TK were co-transfected into TE-1 and KYSE-150 cells. The relative OGT promoter activity was detected by dual-luciferase reporter assay. **(E)** Immunohistochemical staining of Pol ι, OGT and protein O-GlcNAcylation in paraffin-embedded ESCC tissues. Scale bar = 100 μm. **(F)** level of OGT and protein O-GlcNAcylation in ESCC and adjacent tissues based on IHC score. **(G)** The correlation between Pol ι and OGT, and the correlation between OGT and protein O-GlcNAcylation were evaluated based on IHC score in 114 tumor tissue samples. **(H)** Survival analysis based on the IHC score of protein O-GlcNAcylation in 114 ESCC samples. Kaplan–Meier survival analysis was applied. *P < 0.05, **P < 0.01, ***P < 0.001.

Considering the pivotal role of G6PD, the rate-limiting enzyme of PPP, in glucose flux redirection and cancer cell proliferation, we then assessed its activity in Pol ι differentially expressed ESCC cell lines. As shown in **Figure 2D**, the enzymatic activity of G6PD was 2.5-fold higher in Pol ι upregulated TE-1 cells than control cells. On the contrary, its activity dropped to around 75% when Pol ι was knocked down in KYSE-150 cells. Of note, in our subsequent studies, we found that the protein level of G6PD did not change (**Figure 3B**). Consistently, cellular NADPH concentration showed a similar pattern, which jumped to about 150% in TE-1 Pol ι cells while declined approximately 50% in KYSE-150 shPol ι cells (**Figure 2E**).

These results indicated that Pol ι directs the glucose flux to PPP through activation of G6PD.

### Pol ι Promotes G6PD O-GlcNAcylation and Overactivation

Since the enzymatic activity of G6PD is tightly regulated by OGT induced O-GlcNAcylation (30) and OGT was found to be downregulated when Pol ι was knocked down in KYSE-150 cells (**Figure 2B**), we postulated that Pol ι may modulate G6PD activity by regulating OGT expression. As shown in **Figures 3A, B**, both quantitative RT-PCR and Western blot assay indicated that OGT expression is enhanced in TE-1 Pol ι cells while decreased in KYSE-150 shPol ι cells. Moreover, the protein level of G6PD remained unchanged. Subsequently, we detected the O-GlcNAcylation of immunoprecipitated G6PD using an O-GlcNAcylation antibody RL2. As shown in **Figure 3C**, overexpression of Pol ι increased and downregulation of Pol ι decreased the O-GlcNAcylation of G6PD, indicating that OGT induced O-GlcNAcylation of G6PD is regulated by Pol ι. We further assessed the OGT promoter activity using dual-luciferase reporter assay and found that the OGT promoter activity is significantly increased in Pol ι overexpressed TE-1 cells and decreased in Pol ι downregulated KYSE-150 cells (**Figure 3D**).

We also tested the expression of Pol ι, OGT and O-GlcNAcylation in tissues of 114 ESCC patients by IHC. Pol ι and OGT positive staining were mainly found in the cytoplasm, while O-GlcNAcylation positive staining was found in both the nucleus and cytoplasm (**Figure 3E**). The level of OGT and protein O-GlcNAcylation was significantly higher in tumor tissues than that in adjacent tissues (**Figure 3F**). The correlation between Pol ι, OGT and O-GlcNAcylation based on their IHC score was further analyzed. As shown in **Figure 3G**, Pol ι expression was positively correlated with that of OGT (r = 0.48, *P* < 0.001). Similarly, OGT expression was also positively correlated with the level of O-GlcNAcylation (r = 0.43, *P* < 0.001). We then applied Kaplan–Meier survival analysis and found that patients harboring a higher level of O-GlcNAcylation exhibit poor prognosis (*P* = 0.019, **Figure 3H**).

Taken together, these data showed that Pol ι induces OGT expression to promote G6PD O-GlcNAcylation and activation. Moreover, Elevated O-GlcNAcylation correlates with increased tumor size and poor patient prognosis.

### Pol ι Modulates OGT Expression Through the Erk Signaling Pathway

**Figure 4:**
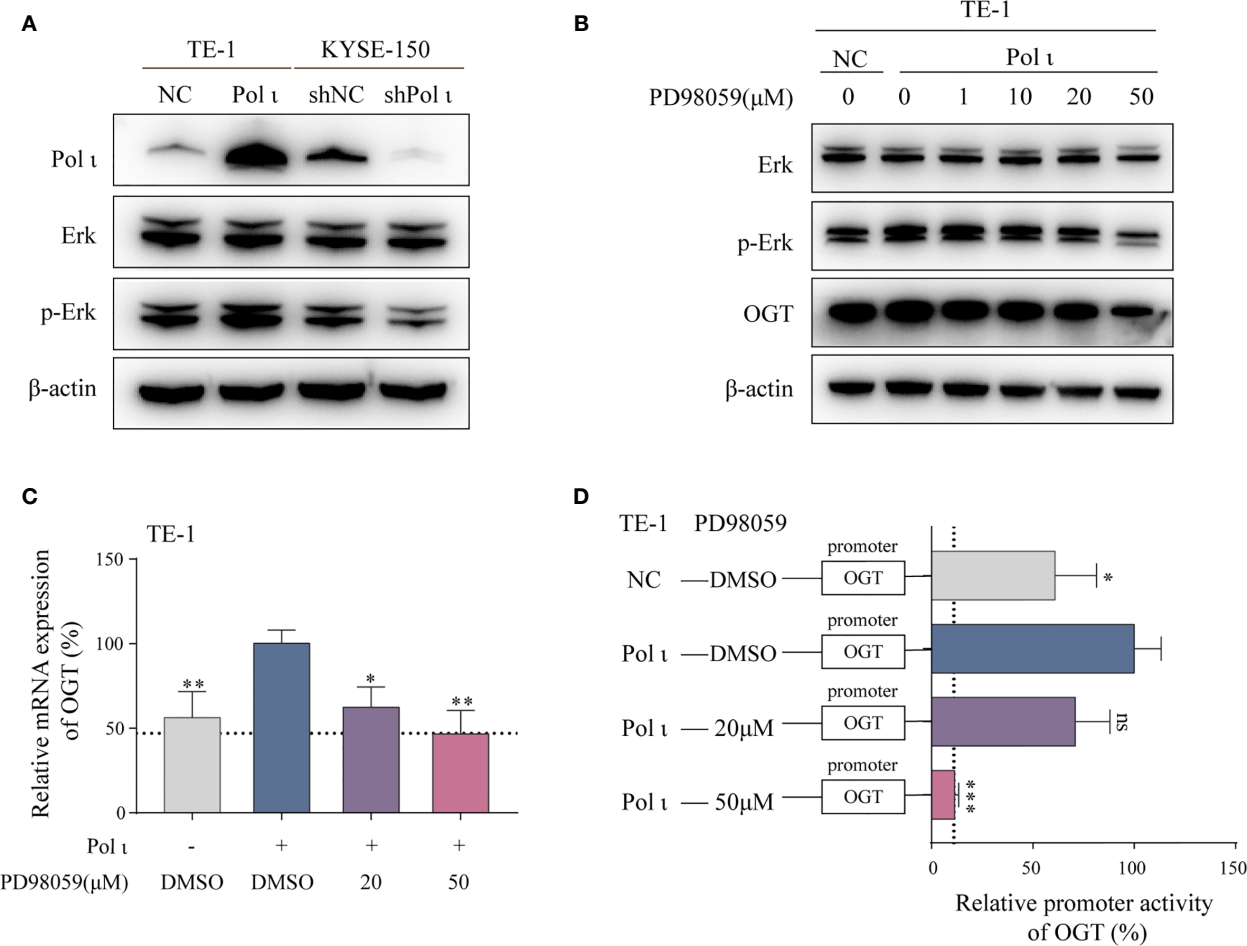
Pol ι regulates OGT expression through the Erk signaling pathway. **(A)** Western blot confirmation of Pol ι expression and Erk phosphorylation. **(B)** PD98059 inhibited Erk phosphorylation and OGT expression in a dose-dependent manner. PD98059 attenuated OGT mRNA expression **(C)** and promoter activity **(D)** in TE-1 cells. *P < 0.05, **P < 0.01, ***P < 0.001.

It has been reported that the Erk signaling pathway is responsible for the transcriptional regulation of OGT (33). Therefore, we evaluated the role of Erk in Pol ι regulated OGT transcription. In line with our previous study, overexpression of Pol ι enhances and knockdown of Pol ι diminishes the phosphorylation of Erk (**Figure 4A**). Subsequently, we used a specific inhibitor PD98059 to inhibit Erk phosphorylation in Pol ι overexpressed TE-1 cell lines. As shown in **Figure 4B**, PD98059 inhibited Erk phosphorylation in a concentration-dependent manner, and decreased OGT protein expression. Moreover, the inhibited Erk phosphorylation was associated with reduced OGT mRNA expression (**Figure 4C**) and OGT promoter activity (**Figure 4D**) in Pol ι overexpressed TE-1 cell lines.

Altogether, these findings indicated that OGT expression is regulated by the Pol ι-Erk signaling cascade.

### Inhibition of G6PD Activity Attenuates Pol ι-Induced Cell Proliferation

**Figure 5:**
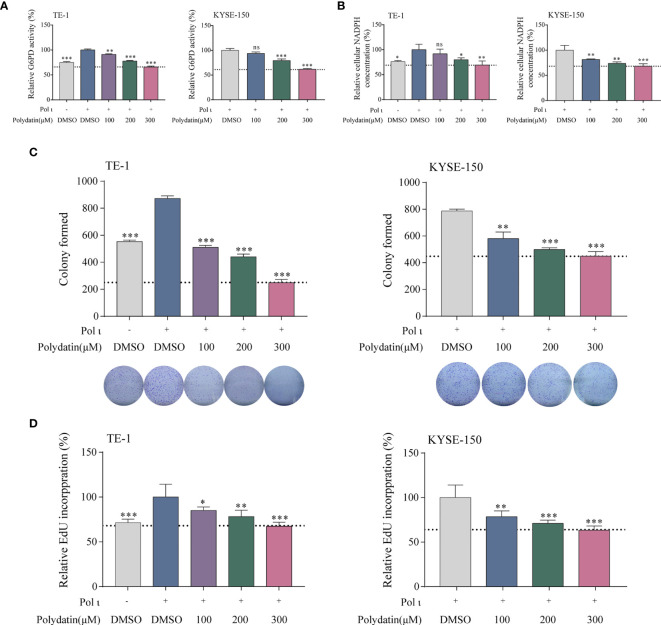
The G6PD inhibitor polydatin suppresses ESCC cell proliferation. G6PD activity **(A)** and cellular NADPH concentration **(B)** decreased in a dose-dependent manner upon polydatin treatment. ESCC cell proliferation after polydatin treatment was assessed by colony formation assay **(C)** and EdU incorporation assay **(D)**. *P < 0.05, **P < 0.01, ***P < 0.001.

To further corroborate the role of G6PD activation in Pol ι-induced ESCC proliferation, a known G6PD inhibitor termed polydatin, a natural molecule found in Polygonum cuspidatum, was used to restrain the G6PD activity in Pol ι overexpressed TE-1 cells and wild type KYSE-150 cells. As shown in **Figure 5A**, we found that the enzymatic activity of G6PD decreases in a concentration-dependent manner in both cell lines once treated with increasing concentrations of polydatin. Similarly, cellular NADPH concentrations were also reduced when higher polydatin concentration was used (**Figure 5B**). We next assessed the influence of G6PD inactivation on cell proliferation. Results from colony formation assay and EdU Cell Proliferation assay indicated that polydatin treatment significantly reduces ESCC cell proliferation compared with the DMSO treated group (**Figures 5C, D**).

Thus, it is obvious that G6PD activity is critical to Pol ι-induced proliferation *in vitro*.

### G6PD Inhibition Attenuates Pol ι-Promoted ESCC Cell Proliferation *In Vivo*

**Figure 6:**
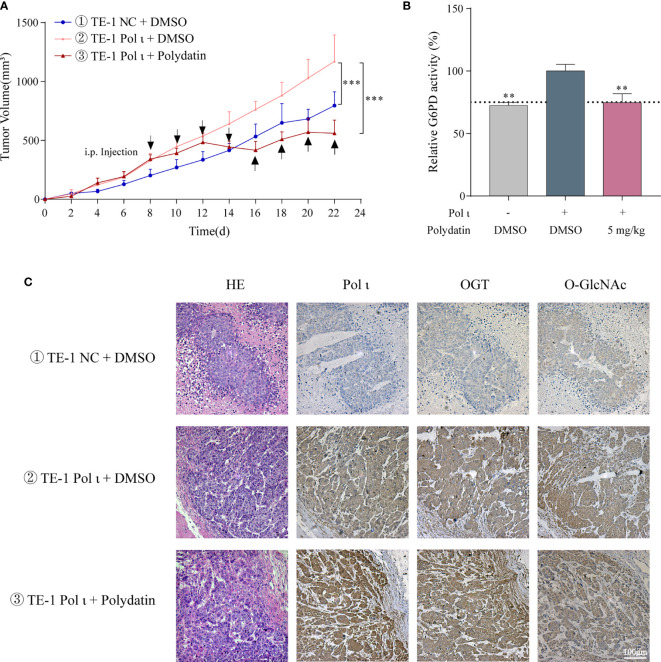
Pol ι promotes ESCC cell proliferation through G6PD activation in *vivo*. **(A)** the tumor volume of xenograft nude mice with different Pol ι expression. Mice were divided into three groups when the average tumor size grew up to 300 mm^3^. 5 mg/kg polydatin was intraperitoneally injected every other day. Same volume of normal saline was used as control. **(B)** relative G6PD activity in tumor tissue. **(C)** hematoxylin and eosin (HE) staining and immunohistochemical staining of Pol ι, OGT and protein O-GlcNAcylation in tumors. **P < 0.01, ***P < 0.001. Scale bar = 100 μm.

We further tested the role of Pol ι and G6PD activation in ESCC cell proliferation *in vivo*. The TE-1 NC and TE-1 Pol ι cells were injected subcutaneously into female nude mice. For drug treatment, 5 mg/kg polydatin dissolved in normal saline was injected intraperitoneally every other day after the average tumor size grew up to 300 mm^3^. Mice in control groups were administrated with the same volume of normal saline. As seen in **Figure 6A**, forced expression of Pol ι promoted tumor growth in TE-1 Pol ι cells comparing with control TE-1 cells, whereas polydatin treatment significantly suppressed Pol ι-induced proliferation of TE-1 cells. Further enzymatic activity assay confirmed the inhibition of G6PD by polydatin in tumor tissues (**Figure 6B**). We then performed IHC to verify the expression of OGT and O-GlcNAcylation. As presented in **Figure 6C**, a concomitant ascending tendency of OGT and O-GlcNAcylation with Pol ι overexpression was evident.

These results demonstrated that G6PD activity is vital for Pol ι-promoted ESCC cell proliferation *in vivo*.

## Discussion

Activation of G6PD plays a pivotal role in cancer proliferation and progression. It shunts glucose flux towards PPP to meet the demands for ribose-5-phosphate and NADPH (17, 18). In this study, Pol ι was found to promote ESCC proliferation by activating G6PD. Pol ι activates G6PD through OGT-mediated G6PD O-GlcNAcylation, and inhibition of G6PD activity by the specific inhibitor polydatin attenuates Pol ι-promoted ESCC cell proliferation both *in vitro* and *in vivo*. Our findings show that Pol ι plays a critical role in ESCC proliferation and progression.

Several lines of evidence indicate that G6PD is a critical effector in Pol ι-promoted ESCC cell proliferation through redirection of glucose flux. First, results from GSEA and seahorse analyzer demonstrated that in KYSE-150 cells, Pol ι downregulation triggers increased oxidative phosphorylation, which is previously reported to impede glucose metabolism and tumor cell proliferation (18). Furthermore, G6PD activity and cellular NADPH concentration are reduced in KYSE-150 shPol ι cells. These results indicate that after Pol ι downregulation, the glucose flux is oriented towards oxidative phosphorylation due to G6PD deactivation. Second, Pol ι upregulation in TE-1 cells results in enhanced G6PD activity and cellular NADPH concentration, suggesting augmentation of glucose flux into PPP. Third, treatment with polydatin, the specific inhibitor of G6PD (34–36), inhibits Pol ι-promoted ESCC cell proliferation both *in vitro* and *in vivo*. These results indicate that G6PD activity is essential for Pol ι-promoted ESCC proliferation.

G6PD overactivation in tumor cells is regulated at the transcriptional or posttranslational level (17, 21, 22, 27–29). As shown in **Figure 3B**, the expression of G6PD remains unchanged in Pol ι differentially expressed cells, suggesting that posttranslational modification may be responsible for G6PD activation, such as OGT induced O-GlcNAcylation (30). Consistent with this assumption, Pol ι upregulation enhances and Pol ι downregulation attenuates G6PD O-GlcNAcylation in our ESCC model systems. It is known that O-GlcNAcylation activates G6PD through enhancing NADP^+^ binding to G6PD and promoting the formation of oligomeric G6PD, leading to increased glucose flux towards PPP (30). As a consequence, cell proliferation and tumor progression are enhanced (17, 19, 29, 30). Therefore, our data indicated that G6PD activation by OGT-induced O-GlcNAcylation promotes ESCC proliferation.

OGT promotes protein O-GlcNAcylation (37, 38) and has been found to be upregulated in most cancers including ESCC (39, 40). In the present study, we found that total O-GlcNAcylation level is correlated with OGT expression and poor patient prognosis (R = 0.43, **Figures 3F, G**). One of the key regulators contributing to OGT overexpression is the hyperactive Erk signaling cascade in cancer (33). The Erk pathway is deregulated in about one-third of human cancers and is one of the key signaling pathways that contribute to cancer proliferation (32). It has been reported that DNA polymerase iota (Pol ι) can interact with p53 (41, 42), which regulates Erk signaling pathway (43–45). Hence, it is possible that Erk signaling pathway is activated by Pol ι *via* the DNA damage repair system.

Therefore, we postulate that elevated OGT may result from Pol ι overexpression in ESCC (14). Our results indicated that the expression of Pol ι is positively correlated with that of OGT in ESCC cells and patient samples (R = 0.48, **Figure 3F**). Furthermore, Pol ι coupled Erk signaling enhances the OGT promoter activity which can be suppressed by PD98059, the MEK signaling specific inhibitor (46). There are four MEK activated MAPK cascades that have been defined: Erk 1/2, c-Jun N terminal kinase (JNK), p38 MAPK and Erk5 (47, 48). It is reported that the MEK inhibitor PD98059 has no effect on the activation of JNK (49) and p38 (50). Otherwise, evidence suggested that PD98059 inhibits both Erk 1/2 and Erk 5 pathways (51). However, it is Erk 1/2 but not Erk 5 activated Elk-1 transcriptional activity (52), which was reported to mediate Erk signaling-induced OGT expression (33). Therefore, in our study, PD98059 induced OGT downregulation is mainly due to its inhibitory effect of Erk 1/2 cascade. Hence, our findings demonstrate that Pol ι enhances OGT expression through the Erk signaling cascade.

However, in the context of Pol ι regulation of OGT expression in ESCC cells, there are still some research gaps to be filled. First, the transcriptional factors responsible for Erk-induced OGT expression in ESCC need to be identified. Second, further scrutiny is required to identify glycosylated proteins in addition to G6PD. Future studies will be carried out to fully understand how Pol ι promotes OGT overexpression and protein O-GlcNAcylation.

In conclusion, the results from the present study demonstrate that Pol ι promotes ESCC proliferation through the Erk-OGT cascade-induced G6PD overactivation. This study provides novel insight into ESCC proliferation and progression, indicating that Pol ι is a potential biomarker and therapeutic target of ESCC.

## Data Availability Statement

The original contributions presented in the study are included in the article/**Supplementary Material**. Further inquiries can be directed to the corresponding authors.

## Ethics Statement

Samples of human ESCC and adjacent tissues were obtained from The Affiliated Suzhou Hospital of Nanjing Medical University (Jiangsu, China) with informed consent. The patients/participants provided their written informed consent to participate in this study. The animal study was reviewed and approved by the Institutional Ethics Committee of Nanjing Medical University.

## Author Contributions

ZS designed the study, performed the experiments, analyzed the data and wrote the manuscript. AG designed the study and performed the experiments. XL performed animal experiments. SZ analyzed the clinical data. CH: designed the study, analyzed the data and wrote the manuscript. JW and JZ supervised the study, reviewed and edited the manuscript. W-QD reviewed and edited the manuscript. All authors contributed to the article and approved the submitted version.

## Funding

The present study was supported by the National Natural Science Foundation of China (81672975, 81802341), the Six Talent Peaks Project of Jiangsu Province of China (WSN095), “333” Project of Jiangsu Province of China (BRA2016071), the Suzhou Administration of Science & Technology (SYS2019091) and the Suzhou Key Medical Center (SZZX201506).

## Conflict of Interest

The authors declare that the research was conducted in the absence of any commercial or financial relationships that could be construed as a potential conflict of interest.
