# Supplementary material for: DNA Polymerase Iota Promotes Esophageal Squamous Cell Carcinoma Proliferation Through Erk-OGT-Induced G6PD Overactivation
Source: Front Oncol. 2021 Jul 20;11:706337. doi: 10.3389/fonc.2021.706337 (PMC8329663; doi:10.3389/fonc.2021.706337)
Supplement: Supplementary file 1 [file DataSheet_1.pdf]

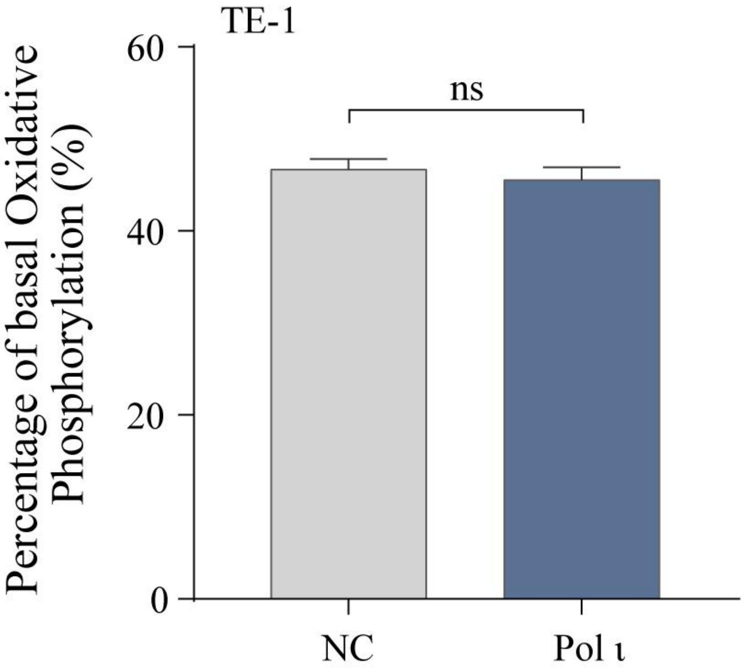

Figure S1. The oxidative phosphorylation level of Pol ι upregulated cells. Rate of cellular metabolism tested by Seahorse analyzer using Real-Time ATP rate assay kit. Total ATP production, the sum of ATP generated from oxidative phosphorylation and Glycolysis, was considered 100%. The ATP production of each metabolic pathway was calculated by oxygen consumption rate (OCR) and extracellular acidification rate (ECAR) after serial injection of oligomycin (1.5 μM) and a mix of rotenone and antimycin A (0.5 μM each).
